# The Dynamic Exome: acquired variants as individuals age

**DOI:** 10.18632/aging.100674

**Published:** 2014-06-16

**Authors:** Jasmin H. Bavarva, Hongseok Tae, Lauren McIver, Enusha Karunasena, Harold R. Garner

**Affiliations:** Virginia Bioinformatics Institute, Virginia Tech, Blacksburg, VA 24061, USA

**Keywords:** Aging, dynamic exome, exome sequencing, personalized genomics, personalized medicine

## Abstract

A singular genome used for inference into population-based studies is a standard method in genomics. Recent studies show that spontaneous genomic variants can propagate into new generations and these changes can contribute to individual cell aging with environmental and evolutionary elements contributing to cumulative genomic variation. However, the contribution of aging to genomic changes in tissue samples remains uncharacterized. Here, we report the impact of aging on individual human exomes and their implications. We found the human genome to be dynamic, acquiring a varying number of mutations with age (5,000 to 50,000 in 9 to 16 years). This equates to a variation rate of 9.6×10^−7^ to 8.4×10^−6^ bp^−1^ year^−1^ for nonsynonymous single nucleotide variants and 2.0×10^−4^ to 1.0×10^−3^ locus^−1^ year^−1^ for microsatellite loci in these individuals. These mutations span across 3,000 to 13,000 genes, which commonly showed association with Wnt signaling and Gonadotropin releasing hormone receptor pathways, and indicated for individuals a specific and significant enrichment for increased risk for diabetes, kidney failure, cancer, Rheumatoid arthritis, and Alzheimer's disease– conditions usually associated with aging. The results suggest that “age” is an important variable while analyzing an individual human genome to extract individual-specific clinically significant information necessary for personalized genomics.

## INTRODUCTION

Aging is a process by which an individual's body changes in its own unique way. Aging proceeds through complex processes involving inherited genetics, the environment to which one has been exposed, and individual status/traits, such as epigenetics [1, 2]. An individual and their discrete tissues may encounter a variety of environmental stressors (chemicals, mutagens, ultraviolet and other radiation exposures) during the course of their life which may enhance biological damage (including oxidative stress), which in turn may cause mutagenesis [1, 3, 4], and contribute to age-associated diseases [5]. Studies of cellular aging have largely focused on telomere shortening. While telomere shortening is a dynamic genomic change, the impact of shortening is apparently minor, perhaps because the genome has built protective and compensatory mechanisms to shield ‘important’ genes from chromosome end erosion [6, 7]. Additionally, gene targeting studies show that telomerase-deficient mice do not age rapidly; in fact, overt phenotypes are not observed for several generations [8]. A recent report describes changes in the exome of a normal single cell and those changes are inherited as the cell propagates [9]. With age, such additive changes in coding regions may promote aging and age related diseases, and result in significant mosaicism. In population-scale studies, however, bulk measurement of an individual genome and the assumption that it completely describes the individual is the preferred approach over sequencing individual cells [10-12]. These studies characterize a variety of important diseases, especially cancer, and ethnic differences found within the population. Recently, three studies reporting exome sequencing for autism spectrum disorder (ASD) compared three very large cohorts of children to the reference genome, which was determined from adult sequences [13-15]. These studies, although characterizing a single disease lacked consensus, perhaps in-part as a consequence of an inappropriate (not age-matched) reference [16].

The inability of genomic variants to fully explain known or suspected inherited and spontaneous components of a wide variety of diseases may indicate that there may be additional undiscovered factors that complicate analysis. These factors include the number and rapidity with which one accumulates genomic variants, which if known could be compensated for, like ethnicity and sex. Genetic characterization of aging, therefore, may hold a key to questions regarding the importance of acquired somatic variants, variation in aging within a population, and their role in human diseases. Adding time and the accompanying mosaic changes as variables may enhance the accuracy and utility of population-scale analysis of human traits and disease. In an attempt to begin to address this gap, we hypothesized that the inherited genome is not static but rather dynamic with time with individual experiences punctuating genomes differently. To test this hypothesis we used exome sequencing of normal epithelial samples from three healthy individuals serially collected at different ages in their life.

## RESULTS AND DISCUSSION

### Single nucleotide variant analysis

We targeted 201,071 exons (62.2 Mb target sequence) in epithelial samples serially collected from three individuals and sequenced them at high coverage (152x, average). Overall, exome enrichment efficiency was 99% (200,264 average target exons sequenced). This enabled 61.6 Mb of target sequence to be analyzed for aging induced exomic changes (SNVs, indels and microsatellites).

**Figure 1:**
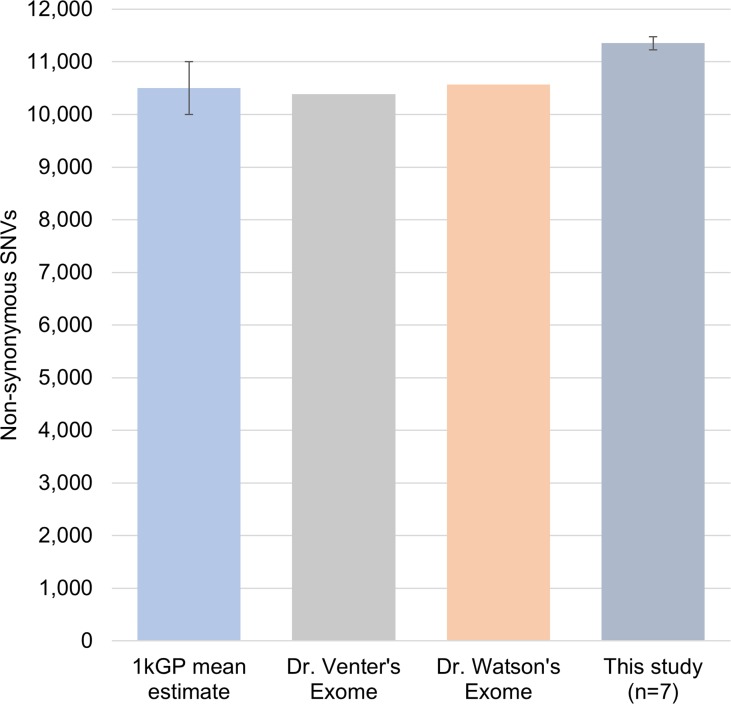
Comparison of number of non-synonymous SNVs Number of non-synonymous SNVs found in samples in our study were comparable with the 1kGP average 10500 ± 500 (n=882), Dr.Venter, and Dr. Watson's exomes. This indicates that the amount of variation identified in the individuals for our study is comparable to previously reported population scale studies.

These individuals had between 11,100 to 11,500 nsSNVs (nonsynonymous single nucleotide variants) at any age time-point studied when compared to the human genome reference (Table S1). The 1000 Genome Project (1kGP) estimated that an individual typically differs from the reference human genome sequence at 10,000-11,000 non-synonymous sites [17]. In other independent studies, Dr. Venter and Dr. Watson's exome were reported to show 10,389 and 10,569 nsSNVs, respectively [18, 19]. These data indicate that the individual's genomic changes in our study are in close agreement with the range of previously reported nsSNVs across individuals (Figure 1), and that this is not a small effect. Using the human genome reference (hg19), we found an overall increase of 725 and 156 SNVs and indels at different ages for two individuals (1 and 2), respectively, and a decrease of 301 and 255 variants in individual-3 upon nine and fifteen years of aging (Table S1), respectively. List of all the variations called is presented in Table S2.

Overall, we found that ~94%, ~ 62%, and ~93% of the variations with respect to the reference genome were present before and after various aging time intervals in individuals 1, 2 and 3, respectively. These suggest the persistent presence of these sites in an individual's specific genome sequence. Thus, although different from the reference genome, as somatic variants, they may not be informative. This sets this study apart from current approaches, which typically would have based the interpretation including all of these variations.

Important to this study is therefore not the number of variants with respect to a (arbitrary) reference genome, rather the differences observed between samples collected from the same individual at different times. To detect the overall frequency of age-specific genomic changes, we examined unique variants in each individual. To avoid false negative results due to inconsistencies in sequenced loci between samples, we report only on those loci completely covered by at least five reads in both samples under comparison. There were 6,005 variants (SNVs and indels) in individual-1 (after 13 years of aging), 45,505 in individual-2 (after 16 years of aging), 5,003 in individual-3 (after 9 years of aging) and 4,948 in individual-3 upon 15 years of aging (Table 1, Table S1).

**Table 1:** Exome variations that differ upon aging in three individuals

	Individual- 1	Individual- 2	Individual- 3	
	(13 years apart)	(16 years apart)	(9 years apart)	(15 years apart)
Total SNVs and indel variations	6,005	45,505	5,003	4,948
Microsatellite variations	173	801	151	164
Synonymous SNVs	604	8455	455	478
Nonsynonymous SNVs	1,239	8,398	904	901
Novel nsSNVs*	849	776	594	576
Stop-gain SNVs**	37	90	15	22
Stop-loss SNVs***	1	8	2	2
Splicing†	3	6	2	0
Frameshift indels	21	128	13	18
Nonframeshift indels	19	165	32	27
Functionally damaging‡	295	1263	207	175
COSMIC variants	107	1609	100	104

* Novel nsSNVs are those not previously reported in the dbSNP 137.

** Stop-gain: A variant that leads to the creation of stop codon at the variant site compared to the reference.

*** Stop-loss: A variant that leads to the elimination of stop codon at the variant site compared to the reference.

† Splicing: A variant within 2-bp of a splicing junction.

‡ Functionally damaging variants are those predicted by Polyphen v2.

**Figure 2:**
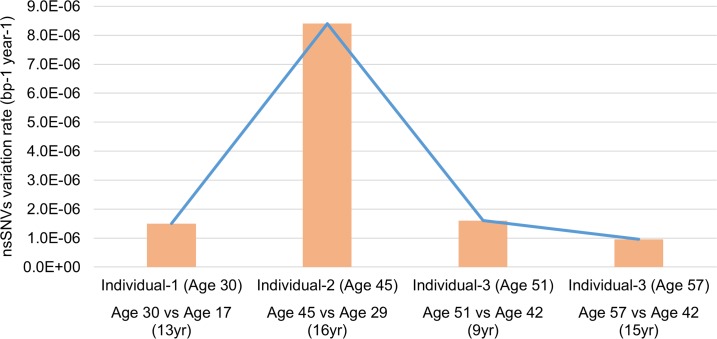
Comparison of nsSNVs variation rate at different ages in three individuals The variation rate varies dramatically between three individuals in our study. This indicates the individual specific genome dynamic of samples in our study.

Exomes of these individuals exhibited a variation rate of 9.6×10^−7^ to 8.4×10^−6^ bp^−1^ year^−1^ for nsSNVs (considering the 62.2Mb of target sequence), which have aged experiencing different stress and environmental exposures and because the genomic region was exclusively exomes may represent a selection pressure that is different from the whole genome (Figure 2). Compared to the reference human genome, individual-2 does not appear very different from other individuals in terms of the total number of genomic variations with respect to the reference (Table S1). However, he exhibits a very high nsSNVs variation rate (8.4×10^−6^ bp^−1^ year^−1^) when referenced to his own previous genomic state. This finding is of particular significance because it highlights the importance of genomic status relative to personal genomics, rather than observations made from a standard reference genome that are missing valuable details especially, individual specific genomic variation trends and behavior.

### Variable indel and microsatellite analysis

Indels that cause frameshifts are usually under negative selection pressure [18]. Indels are the second most abundant type of genetic variation, following single nucleotide substitutions and account for almost a quarter of the genetic variation implicated in disease [20]. As shown in Table S3, we identified a reduction in frame-shift indels and the ratio (frameshift/nonframeshift indels) in older individuals (individual-2 and 3) indicating the negative selection of frameshift indels in these individuals at later age. This is consistent with previous observations [18], and suggests the presence of sustained selection pressure throughout the lifespan of an individual.

Since regions with repetitive sequences exhibit a higher-than-background frequency of indel variation [21], we inspected repeat regions (microsatellites) independently. Repeat containing loci are considered to be highly vulnerable to genomic variation, and they represent as much as 3% of the genome; twice the size of the coding region [22, 23]. We identified 173 variable microsatellites within the 53,161 total microsatellite loci called in both samples from individual-1 (Table 2, S4). This translates to a variation rate of 2.5×10^−4^ locus^−1^ year^−1^ for microsatellite loci, significantly higher than what we measured for nsSNVs. Four of the microsatellite variants were found in exons of known genes (DIAPH1: AGG, RPL14: CTG, BCL6B: CAG and FLJ32682: CCTT), 5' UTR (2) and 3' UTR (27). Three of the four coding variable repeats were nonframeshift.

**Table 2:** Microsatellite variants that differ between samples at different ages and their distributions

	Individual- 1	Individual- 2	Individual- 3	
	(13 years apart)	(16 years apart)	(9 years apart)	(15 years apart)
Total microsatellites called	53,161	49,988	51,902	52,301
Total microsatellites difference	173	801	151	164
% Global microsatellite index	0.3%	1.6%	0.3%	0.3%
**Location of variable microsatellites**				
Exonic	4	26	1	2
Intronic	95	351	80	96
3' UTRs exon	27	224	36	28
5' UTRs exon	2	17	2	4
3' UTRs intron	2	12	4	2
5' UTRs intron	6	37	3	7
Downstream	6	36	4	4
Upstream	8	15	4	5
Intergenic	23	83	17	16

Individual-3 has a comparable number of microsatellite variations to individual-1. Both sets indicate 0.3% of the callable microsatellites vary upon aging. Individual-3 with 9 years of aging shows one acquired variation in an exon (BTN2A1: CCT). This mutation appears to have been acquired between age 42 to 51 with age 57 having the same genotype as age 51. Individual-3 acquired an additional variation in an exon (MEF2A, CAG). It is not clear if this mutation is present at 9 years of aging in this individual as we were not able to genotype this microsatellite locus in the 51 year old sample. The exonic variations found in both of these genes are in frame (Table S4).

Individual-2 has 801 microsatellite variations representing 1.6% of the callable microsatellites upon aging. This is a five times higher microsatellite variation rate compared to the other two individuals (0.3%) in this study, which cannot be attributed to experimental variations for the total number of microsatellite loci called were similar across all samples. The samples for this individual do span the largest amount of time, in total 16 years, though this is not much more than samples from individual-3 that span 15 years. In total there are 26 exonic microsatellite loci found to vary in individual-2 upon 16 years of aging. One of these microsatellites is the same as that found to vary in the samples from individual-3 (MEF2A, CAG). Additionally, one of the exonic microsatellites, which vary in the samples from individual-1, is also indicated to vary in individual-2 (BCL6B, CAG). All the 26 microsatellite loci found to vary in this sample set are in frame. Together, in all three individuals, we identified 33 exomic microsatellite variations of which 32 were nonframeshift. This supports the previous observation that tandem insertions or deletions of repeated motifs is a conservative event (minimizing the number of changed amino acids and the introduction/ elimination of stop codons) [24]. Together, this is the first observation demonstrating persistent effects of selection pressure on indels and repeats among cells in the life span of an individual.

### Biological implications

**Figure 3:**
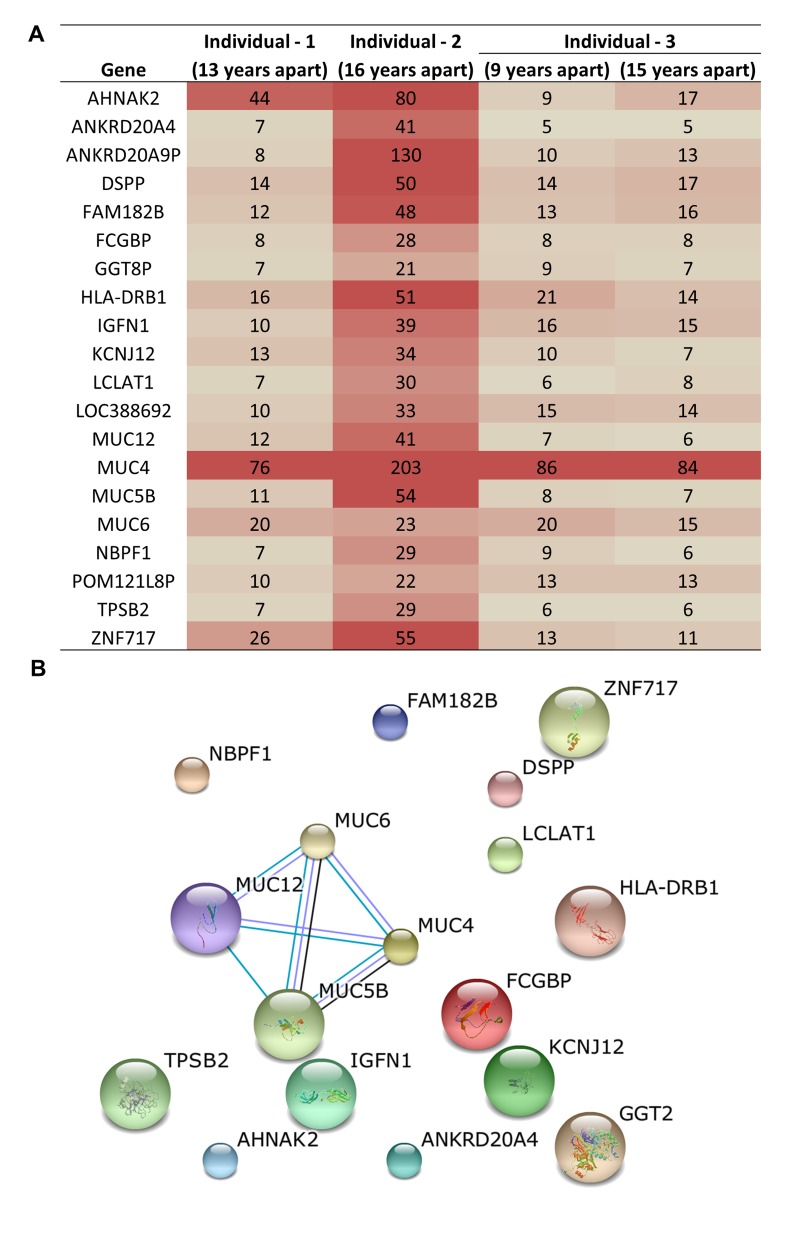
Common genes that acquired genetic variation upon aging and were among the top 100 most frequent variants (**A**) Table shows the number of mutations in each of the genes most frequently impacted in three individuals. (**B**) Protein-protein interaction analysis reveals that for the highly related MUC genes are significant targets for genetic variation in aged samples

Three unrelated and ‘healthy’, individuals demonstrate acquired genetic variants as a consequence of aging and the accumulation of variants occurs at different rates. Mutations were observed in up to 13,000 genes in these individuals including some common genes with consistently high numbers of mutations. To identify the most frequently mutated genes upon aging, we compared the lists of the mutated genes from each individual. This resulted in the identification of 20 genes that are consistently and highly mutated upon aging (Figure 3A). We identified MUC4 as the most frequently mutated gene in all three individuals acquiring >75 mutations. In-fact, four MUC genes (MUC4, MUC5B, MUC6, and MUC12) were among the top mutated genes in all three individuals (Figure 3A). These top 20 genes did not have known protein-protein interactions except for the MUC family genes (Figure 3B). This indicates a previously unknown association among these genes and aging. Previous studies have associated differential MUC4 expression with a number of cancers, including pancreatic, lung, breast, gall bladder, salivary gland, prostate and ovarian cancer, indicating that MUC4 may be a good candidate as a diagnostic and prognostic marker [25]. In one breast cancer study, silencing MUC4 led to reduced expression of HER2, although the molecular mechanism of this interaction is unknown [26]. Over-expression of HER2 occurs in 30% of breast cancers and has been used effectively as an adjuvant therapy drug target in these patients [27]. It is interesting to note here that MUC genes were not previously reported as the most frequently mutated genes in any disease or condition. In addition, the functional significance of the MUC, especially the MUC4 mutations upon aging is unclear at this time and warrants further investigation.

To explore the biological significance of genomic variants in these individuals, we performed pathway enrichment analysis of the affected genes (all genes that showed variations upon aging (Individual-1= 3,138, Individual-2= 13,014, and Individual-3= 2,782 and 2,768 genes, at 9 and 15 years of aging respectively). The PANTHER classification system identified Wnt signaling (involving 5.5% of the total genes on average) and Gonadotropin-releasing hormone receptor (GnRHR) (involving 5% of the total genes on average) pathways with the highest gene associations. Interestingly, it was recently reported that the Wnt signaling pathway enhances the protection of telomeres, and is downregulated in aged skin [28, 29]. The GnRHR pathway is proposed to control central nervous physiology and pathophysiology modulating cognitive changes associated with aging and age-related neurodegenerative disorders [30]. These changes, if found within neurological cells, may indicate the possibility of gradual loss of cognitive function with aging may be due to age-associated genomic modifications.

We subsequently investigated a sub-set of genes that contained damaging (high impact) variants as predicted by Polyphen, and performed disease ontology analysis to find patterns of disease relationship enrichment (Individual-1= 296, Individual-2= 1,267, and Individual-3= 208 and 175 genes, at 9 and 15 years of aging respectively). FunDO (Functional Disease Ontology Annotations) identified genes with a statistically significant (Bonferroni corrected p-value <0.05) disease association for disease ontology terms in these three individuals. Individual-2 showed the highest number of genes enriched for disease associations including genes for diabetes mellitus, cancer, HIV infection, and Rheumatoid arthritis, Individual-1 showed enrichment for diabetes mellitus, leukemia, and cancer (Table 3). Whereas, individual-3 showed the least disease enrichment, except, notably gene variants implicated in mental retardation (Table 3). This analysis revealed the individual specific patterns of disease susceptibility as a direct result of the age associated variants. No metadata are available for these individuals for any correlation with disease history, except that Individual-2 has a history of bone fractures. Osteoporosis is a feature of rheumatoid arthritis and can cause bone loss early in disease [31]. Therefore, because individual-2 is at risk for rheumatoid arthritis, we would have predicted/ expected this individual to be the susceptible to bone fracture. This is one example of possible impactful clinical significance that can emerge from this type of analysis.

**Table 3:** Disease ontology analysis of genes with functionally damaging variants indicates increased risk of developing age-related diseases.

Individual- 1	Individual- 2	Individual- 3	
(13 years apart)	(16 years apart)	(9 years apart)	(15 years apart)
Diabetes mellitus (11)	Diabetes mellitus (31)	Long QT syndrome (2)	Adenovirus infection (3)
Leukemia (10)	Breast cancer (32)		Mental retardation (3)
Kidney failure (5)	Prostate cancer (28)		
Colon cancer (8)	Neoplasm metastasis (17)		
Cancer (13)	Cancer (40)		
Alzheimer's disease (6)	Rheumatoid arthritis (21)		
	Melanoma (14)		
	HIV infection (12)		
	Colon cancer (19)		
	Leukemia (20)		
	Chronic simple glaucoma (5)		
	Atherosclerosis (15)		
	Cirrhosis (6)		

A sub-set of genes, which contained predicted damaging variants (by Polyphen v2) were analyzed using Functional Disease Ontology Annotations (FunDO). The significance of each disease association is evaluated by Fisher's exact test, and diseases that showed Bonferroni corrected p-value <0.05 are considered as significant. Numbers in parenthesis indicates the number of genes associated with disease.

There are additional examples of associations with disease via genes in which there were frequent variants. Recently, a melanoma sequencing study revealed frequent mutations in PREX2 (phosphatidylinositol-3,4,5-trisphosphate-dependent Rac exchange factor 2) as a consequence of ultraviolet exposure [11]. We found that, with age, individual-1 acquired a PREX2 mutation (Chr8:69143713, delT) in the 3' UTR and Individual-2 acquired five mutations (four are synonymous and one deletion). Individual-3 acquired a PREX2 mutation identical to Individual-1 upon nine years of aging, however that variation was not seen in the sample taken at 15 years. This suggests the possibility that the PREX2 mutation, while it may be acquired with ultraviolet exposure, is negatively selected in this individual probably to protect from the pathogenesis of skin cancer. Environmental exposure selects for genomic changes for better adaptation and to increase fitness, a process that usually spans several generations [32]. With the relatively longer and growing life spans of humans and the spectrum of stressors to which humans are exposed, it is very likely that individual cells and tissues may exhibit genomic changes shaped by local selective forces within the life span of an individual. This finding further suggests the influence of environmental exposures on the genome with aging and importance of serial measurements to monitor genome dynamics. Therefore, it will be of interest to establish measures to quantify individual genome dynamics with age and with tissue specificity, which may help detect the rate of functional decline, the vulnerability of an individual to age-related diseases, and potentially predict longevity.

Previous hypotheses on molecular damage and aging suggested that, 1) molecular damage limits life span not because of cellular decline but because of decline in cellular robustness, 2) although, the accumulation of molecular damage is observed, it is not the driving force for aging, and 3) mutations are not random, they are selected to activate/ inactivate particular pathways [33]. In this study, we observed that the genome dynamic, influenced by aging itself or environmental factors, could influence pathogenesis, and might initiate organ or system level damage that are frequently observed upon aging and in age related diseases in humans. Environmental exposures are known to influence the genome in a way that may increase the fitness or induce deleterious mutations [32, 34]. Therefore, age, genome dynamic and environmental exposures are interlinked and together could result in sustained selection and/or accumulation of variations and/or non-mutational hyper-activation of signaling pathways involved in aging. This could reduce overall robustness at a cellular level and cause degeneration of cells, tissues, and organ systems. Although, it is arguable to define aging as a consequence of molecular damage or *vice versa* at this time, both are positively correlated.

Taken together, the exome sequencing of individual's genome upon aging indicates that implementation of personalized genomics health strategies will require more thorough and potentially continuous analysis of individual's genomes to optimize outcomes. These data demonstrate that the exome of an individual is dynamic and constantly experiences environmental and evolutionary pressures and over time enriches for deleterious variants. This finding indicates that the accumulation of somatic variants and possibly the rate of accumulation will contribute to how an individual ages, and prompting age-related diseases. It challenges our existing approach in population-scale sequencing studies and establishes “age” as an important variable that must be accounted for in the analysis and interpretation of any given human genome. These observations are supportive of new paradigm, “Multiple genomes per individual”.

## METHODS

### Sample details

DNA from primary skin fibroblasts was obtained from the Aging Cell Repository, NIA at the Coriell Institute (Camden, NJ). These samples were serially collected from three Caucasian male individuals at different time point in their life: Individual-1 (age 17- AG06234 and age 30-AG13153), Individual-2 (age 29-AG05415 and age 45-AG13353) and Individual-3 (age 42-AG05416, age 51-AG11364 and age 57-AG13145) (Total numbers of samples were seven).

### Library construction and Exome enrichment

DNA-seq libraries were constructed using Illumina's TruSeq DNA Sample Preparation Kit-Set A/B (P/N FC-121-2001/2002). Briefly, 1.5 μg DNA was fragmented using Covaris M220 to 400bp. A gel-free method recommended in the protocol was used to prepare the library. The ends were repaired and a ‘A’ base added to the 3', which prepares the DNA fragments for ligation to the adapters that have a single ‘T’ base overhang at their 3' end. The adapters enable PCR amplification and hybridization to the flow cell. The library generated was validated using Agilent 2100 Bioanalyzer and quantitated using Quant-iT dsDNA HS Kit (Invitrogen; Carlsbad, CA). Exome enrichment was performed using TruSeq Exome Enrichment Kit (FC-121-1024; Illumina). Samples were pooled at concentrations of 500 ng each and enriched following the manufacturer's standard protocol. Enriched samples were quantitated based on Quant-iT dsDNA HS Kit (Invitrogen) and qPCR.

### Cluster Generation and HiSeq Sequencing using RapidRun Mode

Libraries were clustered onto a flow cell using TruSeq^®^ Rapid PE Cluster Kit – HS (PE-402-4001), and sequenced 2X for150 cycles using TruSeq^®^ Rapid SBS Kits – HS (FC-402-4001) on HiSeq 2500^®^. Reads that passed the Illumina chastity filter were kept. Reads passed the chastity filter if they had, within the first 25 cycles, no more than one cycle of a chastity below 0.6 (Chastity = Highest intensity/(Highest intensity + Next highest intensity)). An average of 112.2 million high quality 150bp reads (passed Chastity filter) were generated from exome-enriched samples equivalent to 16.8 billion DNA bases per exome. We opted for longer (400bp) DNA fragments for library preparation and longer read length (150bp) for sequencing to enhance the quality and results, especially within repeat regions.

### Variant discovery and quality control

The exome enrichment kit targeted 201,071 exons equivalent to 62.2 Mb target sequence in these epithelial samples serially collected. The read sequences were aligned to hg19 with BWA [35] resulting 110.6 million average reads mapped to hg19 per sample. Exome enrichment efficiency was 99%, as measured by the number of target exons sequenced (average 200,264) out of the total target exons (201,071) per sample. 62.8 million reads mapped on target providing 152x average depth of coverage. Reads were locally realigned around Indels, and raw variants (Single nucleotide variants and Indels) were called using GATK Unified Genotyper [36, 37].

We filtered variants with minimum read depth of ≥5x and mapping quality >30 for high confidence true positive variant calls. In addition, next-generation sequencing has overall very high true positive rate for identified SNVs [38]. In our test using Sanger sequencing, we validated 5 out of 6 loci for true positives. To minimize false negatives, we used a custom Perl script. The script verified loci of age specific variants for the presence of supporting sequence reads (≥5) in both comparison samples. Further, this study complies with the proposed standardization criteria for NGS studies as all samples were uniformly sequenced with identical protocols, sequencing instruments, and the data analysis criteria [16]. Together, these criteria ensure that variant identification is of very high confidence minimizing, false positive and false negative variants as well other technical variability.

### Microsatellite variation and quality control

After aligning the reads to hg19 with BWA version 5.10, we applied our microsatellite genotyping software, requiring a minimum of 15 reads completely covering a loci in order to call a genotype for each sample at these challenging loci that require more stringency [39, 40]. This software was recently updated to accept hg19 alignments by converting the prior microsatellite coordinate using the UCSC Genome Lift-Over tool [41]. The software was also updated to speed up the sub-functions allowing us to run an exome-sequenced sample in under 3 hours on a single core of an Intel Xeon 5500/5600 processor. We performed tests between our original hg18 software and the new, faster hg19 version to determine if there are any microsatellites calls that differed. We identified 530 microsatellites for which different genotypes were obtained. These microsatellites were removed from our analysis set leaving at most 856,104 callable microsatellite loci per sample. We were able to call on average 51,769 microsatellite loci from the exome-enriched samples with annotations. We used the locations of Refseq genes obtained from the UCSC Table Browser to determine the relative position of the called microsatellites with respect to genes [42]. We have previously validated that our microsatellite-based genotyping method has 96.5% accuracy [40].

### Gene annotation, disease enrichment, and functional impact analysis

All identified variants (SNVs and indels) were annotated using ANNOVAR package using Reference Gene [43]. Splice site variants were identified as occurring within two base pairs of any intron/exon boundary. Variants that created a stop codon at the variant site were considered as stop-gain variants. Variants that eliminated stop codon at the variant site were considered as stop-loss variants. All identified variants were annotated for a variety of characteristics and analyzed. Single Nucleotide Polymorphism database (dbSNP 137) was used to check novel variants. Polyphen 2.0 was used to predict the functional impact of non-synonymous variants [44]. The Catalogue of Somatic Mutations in Cancer (COSMIC) database v64 was used to identify somatic cancer variants. Ingenuity Pathway Analysis (IPA) was used for canonical pathway enrichment analysis. The Functional Disease Ontology (FunDO) used for disease enrichment analysis [45].

## SUPPLEMENTARY INFORMATION AND TABLES


