# Supplementary material for: The Dynamic Exome: acquired variants as individuals age
Source: Aging (Albany NY). 2014 Jun 16;6(6):511–21. doi: 10.18632/aging.100674 (PMC4100812; doi:10.18632/aging.100674)
Supplement: Supplementary file 1 [file aging-06-511-s001.pdf]

SUPPORTING INFORMATION

Please browse the full text version of this manuscript to see the Supporting Tables S2 and S4.

Table S1. Statistics on all genetic variants called with respect to the human genome reference (hg19) and their characteristics and biological predictions.

	**Individual - 1**		**Individual - 2**		**Individual - 3**		
	Age 17	Age 30	Age 29	Age 45	Age 42	Age 51	Age 57
Read coverage on target	139x	168x	137x	143x	164x	159x	153x
Total SNV and indels vs hg19	61,892	62,617	61,411	61,567	61,892	61,591	61,637
Variable microsatellites vs hg18	1,425	1,612	1,300	1,282	1,374	1,351	1,335
**Exonic function (Reference Gene)**							
nonsynonymous SNVs	11,353	11,498	11,100	11,331	11,427	11,383	11,382
synonymous SNVs	11,658	11,797	11,709	11,736	11,718	11,685	11,670
frameshift indels	139	139	121	154	116	120	116
nonframeshift indels	202	207	221	217	231	234	234
stopgain	100	110	93	86	80	73	78
stoploss	12	13	14	10	15	13	15
**Reference Gene function**							
Upstream	29	28	36	34	27	30	31
Downstream	42	45	36	39	33	37	37
Exonic	24,001	24,287	23,779	24,055	24,096	24,022	23,997
Intergenic	250	251	263	269	296	300	294
Intronic	228	233	238	236	258	241	255
Splicing	40	43	49	49	47	50	47
3' UTRs	27,430	27,703	26,631	26,234	26,487	26,325	26,358
5' UTRs	3,722	3,828	4,415	4,417	4,502	4,506	4,499
**Databases match**							
COSMIC somatic cancer mutation database	2,057	2,068	2,071	2,035	2,129	2,124	2,120
nsSNVs not reported in dbSNP137	934	1,053	690	671	820	750	740
**Functional effects prediction**							
Polyphen: Probably damaging (D)	1,150	1,224	1,156	1,182	1,227	1,190	1,181
Mutation tester: Disease causing automatic (A)	29	35	25	23	30	23	27

Table S3. Disease ontology analysis of genes with functionally damaging variants indicates increased risk of developing age-related diseases.

	**Individual - 1**	**Individual - 2**	**Individual - 3**	
	**(13 years apart)**	**(16 years apart)**	**(9 years apart)**	**(15 years apart)**
Nonframeshift indels	19	165	32	27
Frameshift indels	21	128	13	18
Ratio	1.11	0.78	0.41	0.67
